# Unraveling the Metabolic Mechanisms and Novel Biomarkers of Vulvar Lichen Simplex Chronicus Using Skin Biopsy and Tape Stripping Samples

**DOI:** 10.3390/metabo15090566

**Published:** 2025-08-22

**Authors:** Tian He, Fanrui Xu, Jing Liang, Qing Feng, Dan Cheng, Linlin Xiao, Maoyu Liu, Xuerui Zhang, Xin Wang, Yang Yang, Dan Zhu, Sergey Tumanov, Richard D. Cannon, Ting-Li Han, Shufang Chang

**Affiliations:** 1 Department of Obstetrics and Gynecology, Second Affiliated Hospital, Chongqing Medical University, Chongqing 400010, China; 2023130211@stu.cqmu.edu.cn (T.H.); 2024110795@stu.cqmu.edu.cn (F.X.); 305467@hospital.cqmu.edu.cn (J.L.); 303571@hospital.cqmu.edu.cn (Q.F.); 305049@hospital.cqmu.edu.cn (D.C.); rachelxiao@hospital.cqmu.edu.cn (L.X.); 306218@hospital.cqmu.edu.cn (M.L.); 305124@hospital.cqmu.edu.cn (X.Z.); danzhu750@gmail.com (D.Z.); 2 State Key Laboratory of Ultrasound in Medicine and Engineering, College of Biomedical Engineering, Chongqing Medical University, Chongqing 400016, China; 2021110749@stu.cqmu.edu.cn; 3 Mass Spectrometry Centre of Maternal-Fetal Medicine, Chongqing Medical University, Chongqing 400016, China; 2h7593@hospital.cqmu.edu.cn; 4 Department of Obstetrics, The First Affiliated Hospital of Chongqing Medical University, Chongqing 400016, China; 5 Heart Research Institute, Newtown, NSW 2042, Australia; sergey.tumanov@sydney.edu.au; 6 Department of Oral Sciences, Faculty of Dentistry, Sir John Walsh Research Institute, University of Otago, Dunedin 9054, New Zealand; richard.cannon@otago.ac.nz

**Keywords:** vulvar lichen simplex chronicus (VLSC), tape stripping, skin biopsy, metabolomics, TRPV1, 20-HETE

## Abstract

Background/Objectives: Lichen simplex chronicus (LSC) of the vulva is a chronic dermatologic disorder characterized by persistent pruritus, compulsive scratching, and progressive thickening of the vulvar skin. Currently, LSC diagnosis primarily relies on clinical presentation, with histopathological examination performed when the diagnosis is unclear. However, the precise pathogenic mechanisms driving the disease remain poorly understood. This study aimed to investigate the pathogenesis of LSC and evaluate the feasibility of tape stripping as a non-invasive diagnostic technique. Methods: Skin specimens were obtained using both traditional biopsy and tape stripping methods, and the metabolites and oxidized lipids in these samples were analyzed using advanced mass spectrometry techniques. Results: Our findings suggest that 20-hydroxyeicosatetraenoic acid (20-HETE), an oxidized derivative of arachidonic acid (AA), activates the TRPV1 receptor, thereby exacerbating the itch–scratch cycle. This activation upregulates energy metabolism and promotes epidermal hyperplasia, providing new insights into the disease’s pathophysiology. Conclusions: Our study suggests that tape stripping could serve as a viable non-invasive diagnostic tool for LSC, with linoleic acid (LA) and AA potentially acting as biomarkers for the disease.

## 1. Introduction

Lichen simplex chronicus (LSC) of the vulva is a dermatological disease characterized by the process of lichenification, which involves the thickening and hardening of the skin due to persistent itchiness and scratching [1]. The prevalence of vulvar LSC has been reported to reach up to 1.7% in the general population but exceeds 10% in vulvar specialty clinics [2,3,4]. The symptoms of LSC are typically attributed to a persistent itch–scratch cycle, whereby scratching worsens chronic itchiness, causing prolonged skin changes and sustained discomfort [5]. In severe cases, excoriation or skin abrasion also occurs [5]. Visual inspection of lichenification and itching symptoms have traditionally been the criteria for diagnosing LSC, while skin biopsies are recommended for clarification in cases of diagnostic ambiguity [6]. However, recent studies have often overlooked the significance of elucidating the underlying mechanisms contributing to LSC and identifying biomarkers, despite the fact that this knowledge could facilitate diagnostic precision and inform personalized treatments.

Diagnosis of atypical LSC traditionally relies on invasive biopsy procedures to provide detailed histological insights into skin changes [5]. While biopsies generate extensive information, their invasive nature raises patient concerns regarding pain and potential harm, posing diagnostic challenges and impacting post-treatment follow-up [7]. Currently, biopsy is the main procedure used to diagnose LSC; no non-invasive techniques are widely employed for this purpose [8]. In recent years, tape stripping has emerged as a non-invasive technique, particularly for studying epidermal metabolites in various skin conditions [9]. This technique involves the application of adhesive tape on the skin surface to collect metabolites from the stratum corneum and skin secretions, providing a simplified and minimally invasive sampling method. Despite its widespread use in skin metabolomic studies [10], tape stripping has not yet been applied to collect metabolic profiles from vulvar skin. Exploiting the non-invasive benefits of tape stripping enables a less invasive and more efficient collection of vulvar skin metabolites.

The persistent itch–scratch cycle is believed to be the main cause of lichenification in LSC [11]. Injurious stimuli, such as friction and scratching, can compromise the skin barrier, allowing itch-inducing molecules to infiltrate the LSC epidermis, thereby eliciting itching [11]. These pruritogenic molecules bind to specific receptors on cutaneous nerve fibers, initiating neuronal activation and transmitting itch signals to the spinal cord [11]. The nonhistaminergic itch in LSC is mediated by the G protein-coupled receptors (GPCR) and/or ion channels, especially the transient receptor potential (TRP) channels [12,13]. Recent research has indicated that itching could be alleviated by activating the TRPV channels [14]. However, the identity of the itch-inducing molecules responsible for LSC remains elusive. Recent findings suggest that steroids target enzymes such as phospholipase A2 (PLA2) to mitigate itch by modulating the lipid metabolism and exerting anti-inflammatory effects. Analogous to LSC, atopic dermatitis (AD) is characterized by persistent itching, with increased itch associated with alterations in lipid metabolism, notably involving arachidonic acid (AA) and corticosteroids [15,16,17]. Andoh et al. discovered that oxylipins, fatty acid peroxidation catabolites, are implicated in the pruritus observed in AD in murine models [18]. Patients with severe itching associated with AD often exhibit significant skin thickening [19]. The persistent itch–scratch cycle can exacerbate skin thickening and lichenification, yet the specific pruritic-inducing molecules and the underlying metabolic alterations involved in the itch–scratch cycle of LSC patients remain unknown.

In this study, we compare the metabolic profiles derived from skin biopsies and tape-stripped samples of patients with LSC using mass spectrometry-based metabolomics to elucidate the underlying metabolic mechanisms in LSC [20], with the potential to guide future diagnostic and therapeutic strategies.

## 2. Materials and Methods

### 2.1. Study Participants

The initial cohort for this study comprised 36 female patients diagnosed with localized LSC. These patients were recruited from the gynecology outpatient clinic at the Second Affiliated Hospital of Chongqing Medical University during the period from 2021 to 2022. Metabolic data collected from these patients were employed to construct a predictive model using machine learning techniques. For independent validation of the constructed model, an additional cohort of 11 patients was subsequently recruited from the same clinic. Samples were collected from each participant using both tape stripping and biopsy procedures. From each individual, samples were obtained from the lesional skin, as well as from the adjacent normal skin, categorizing them into four distinct groups: lesional-skin tape stripping (LSC Tape), normal-skin tape stripping (NOR Tape), lesional-skin biopsy (LSC Biopsy), and normal-skin biopsy (NOR Biopsy). The LSC lesions were assessed according to the criteria proposed in 1990 by the International Society for the Study of Vulvar Disease [21]. The inclusion criteria were as follows: (1) Patients who were >18 years of age and (2) clinical diagnosis of LSC based on medical history, clinical symptoms, physical examination, and necessary auxiliary examinations. Exclusion criteria included (1) biopsy results suggesting vulvar cancer or vulvar intraepithelial neoplasia; (2) acute or active vulvar/vaginal infection; (3) pregnant, lactating, and menstrual women; (4) physical therapy modalities including high-intensity focused ultrasound (HIFU) for the vulva, fractional CO_2_ laser treatment, and any history of glucocorticoid treatment within three months prior to biopsy; (5) the use of immunosuppressive drugs, chemotherapy, anti-inflammatory drugs, antihistamines, or steroids within three months; (6) serious heart, liver, or kidney diseases or severe diabetes; and (7) recent use of vaginal cleansers.

All procedures performed in this study were in accordance with the principles of the Declaration of Helsinki, 1964. The study was approved by the ethics committee of the Second Affiliated Hospital of Chongqing Medical University (Ethic #: 2021826). Written informed consent was obtained from all participants included in the study at enrollment.

### 2.2. Clinical Information

Basic information (age, BMI, reproductive history, and disease duration) was collected from the electronic medical records. To avoid subjectivity and variability in assessments, this study employed Cattaneo clinical symptom and sign score to evaluate the intensity of itching, skin elasticity, color, and lesion area on a scale from 0 (none) to 3 (severe) for each parameter [22] (Table S1). Lesion areas were quantified as a percentage of the total perineal area, with higher scores denoting increased severity. The overall severity of the condition is determined by the cumulative Cattaneo scores, which sum the individual scores for the evaluated clinical symptoms and signs.

### 2.3. Tape and Skin Biopsy Sample Collections

**Figure 1:**
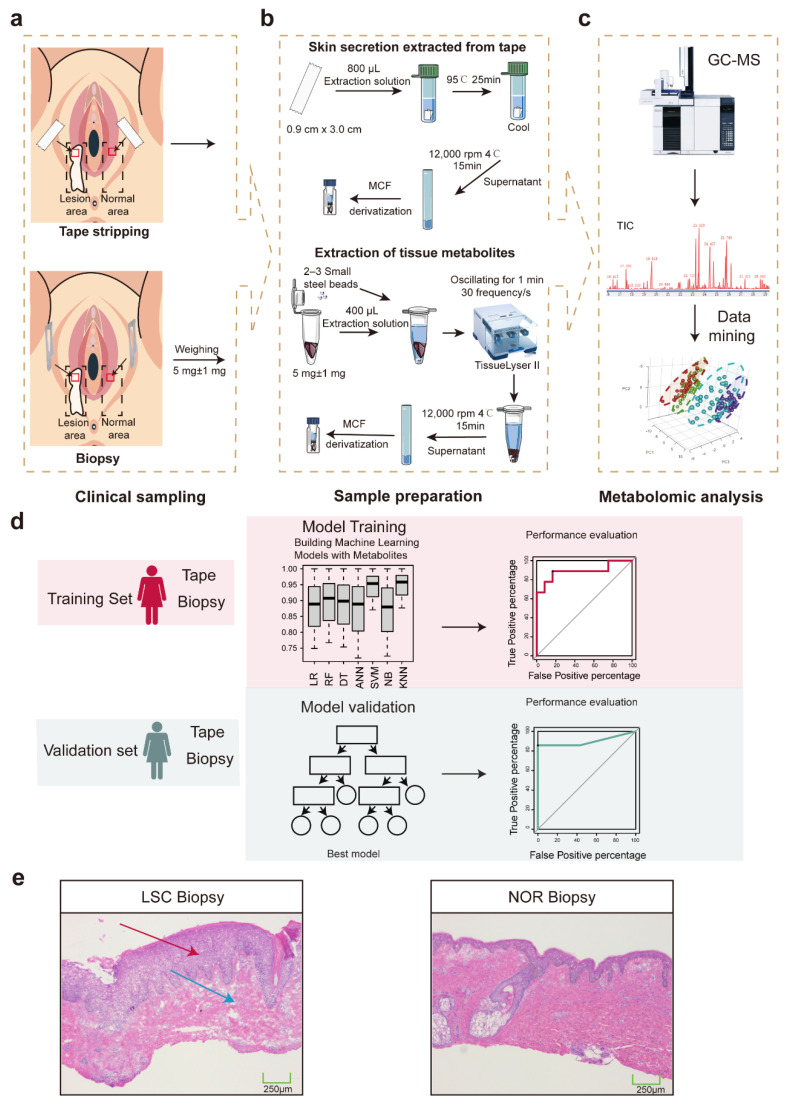
Flowchart of experiment design. (**a**) Skin biopsy and tape stripping sampling. (**b**) Skin metabolite extraction. (**c**) GC–MS-based metabolomics approach and metabolite data mining. (**d**) Schematic overview of machine model construction using training (red) and validation (blue) sets of data. (**e**) Histological sections of the vulvar lichen simplex chronicus skin (LSC Biopsy) and normal vulvar skin (NOR Biopsy) with H&E. Red arrows indicate epidermal thickening in the granular layer and stratum spinosum; blue arrows denote chronic inflammatory infiltration in the superficial dermis. Abbreviations: MCF, methyl chloroformate; GC–MS, gas chromatography–mass spectrometry; ANN, artificial neural network; DT, decision tree; KNN, K nearest neighbor; LR, logistics regression; NB, Naïve Bayes; RF, random forest; SVM, support vector machine; LSC, lichen simplex chronicus; and NOR, normal.

The overall experimental design is summarized in Figure 1a. Firstly, adhesive tape (0.9 cm × 3.0 cm) was placed onto the lesion or on adjacent normal skin and held in place for 15 s. For skin biopsy, a 3 mm × 3 mm × 2 mm sample of skin was excised from both the lesion and adjacent normal skin. A scalpel was used to allow for a larger excision, facilitating both biopsy tissue collection and subsequent metabolite analysis for diagnostic purposes. Specimens were then stored at −80 °C. To clarify, a total of four samples were collected from each participant: one tape-stripped and one biopsy sample from the lesional area and one tape-stripped and one biopsy sample from adjacent normal skin. All sampling sites were localized to the inner aspect of the labia majora within the perineal region.

### 2.4. Hematoxylin–Eosin (H&E) Staining for Skin Biopsy

Following the collection of skin biopsies, samples were excised, cleansed, and fixed in a 10% formalin solution at 4 °C for 24 h. After fixation, the samples underwent dehydration, were embedded in paraffin, and were sectioned into 5 µm slices. These sections were then stained with Hematoxylin–Eosin (H&E) and examined using an NLCD500 digital biological microscope (Jiangnan Yixin, Nanjing, China).

### 2.5. Sample Preparations for Skin Biopsy and Tape Stripping Specimen

Vulvar biopsy tissue (~5 ± 1 mg) was weighed and placed in a 1.5 mL EP tube containing three magnetic beads and 400 µL of extraction solution (1:1 ratio of 1 M sodium hydroxide and methanol, 0.12 mM D4-alanine, and 0.12 mM D5-phenylalanine), followed by 1 min, 30 frequency/s oscillation in a tissue lyser (TissueLyser II, Qiagen, Hilden, German). Then, the supernatant was isolated by centrifugation at 12,000 rpm (4 °C) for 15 min and transferred to a salinized tube prior to derivatization. The adhesive tape was placed in a 2 mL screw cap tube containing 800 µL of extraction solution (1:1 ratio of 1 M sodium hydroxide and methanol, 0.12 mM D4-alanine, and 0.12 mM D5-phenylalanine), followed by heating in a water bath for 25 min at 95 °C. Then the supernatant was isolated by centrifugation at 12,000 rpm (4 °C) for 15 min and transferred to a salinized tube prior to derivatization.

### 2.6. Quality Control (QC) and Randmomization

Twenty-seven quality control (QC) samples were prepared by pooling portions of the extracted supernatants and subsequently aliquoting the mixture into new tubes. These aliquots were utilized for two distinct experimental procedures: MCF derivatization and lipid oxidation studies. For every set of fifteen samples analyzed, one QC spectrum was acquired to ensure methodological consistency. The randomized ordering of the analysis of the samples was facilitated by the RANK function in Excel, guaranteeing an equal distribution of samples from the normal and abnormal groups for each pair of QC samples.

### 2.7. Methyl Chloroformate (MCF) Derivatization and Gas Chromatography–Mass Spectrometry (GC–MS) Analysis

All extracts were subjected to chemical modification by methyl chloroformate (MCF) derivatization to reduce their boiling points, following the methodology described by Smart et al. [23]. These volatile compounds were subsequently separated using a DB-1701 gas chromatography capillary column (20 m × 180 μm id × 0.18 μm column, Agilent, Santa Clara, CA, USA) and detected by a chromatography–mass spectrometry (GC–MS) system (Agilent Intuvo 9000-5977B) employing electron impact ionization at 70 eV. Operational parameters for the GC–MS analysis were according to previously published research [24]. The system’s inlet temperature was maintained at 300 °C in a pulsed splitless mode, with a helium carrier flow rate of 1 mL/min. Temperatures for the guard chip, auxiliary heater, MS quadrupole, and MS source were set at 300 °C, 300 °C, 230 °C, and 150 °C, respectively. Mass detection ranged from 38 μm to 550 μm, with a scan speed of 2.9 m/s, and the mass spectrometry detector was activated after 4.5 min.

### 2.8. Metabolite Identification

The chromatographic characteristics of metabolites were deconvoluted and identified using Automated Mass Spectral Deconvolution & Identification System software. The metabolites were confirmed by matching both the in-house MCF library spectra > 85% and their respective GC retention times within a 30 s window. The identification of the remaining compounds was performed using a commercial National Institute of Standards and Technology (NIST) mass spectral library [25].

### 2.9. Quantification of Metabolite Concentration

Amino acids, fatty acids, and lactic acids were quantified using chemical standards. Levels of these metabolites were first normalized against the appropriate internal standard and then quantified to absolute concentration using calibration curves obtained with the corresponding chemical standard (five-concentration range from 0 to ~55.4 mM).

### 2.10. GC–MS Data Mining and Normalization

The relative concentrations of metabolites were determined using an R-based script, MassOmics, which calculates peak heights of the most abundant fragment ions within specific retention times [26]. Background contamination and metabolite carryover were mitigated by subtracting the values obtained from blank samples. To enhance quantification, the identified compounds’ concentrations were normalized using two internal standards (d4-alanine and d5-phenylalanine), based on their correlation with metabolites in QC samples [27]. In addition, median centering of QC samples was implemented to compensate for daily batch effects. Dilution factors were corrected using the total ion chromatogram (TIC) method, and for vulva biopsy samples, concentrations were adjusted according to the sample weights.

### 2.11. Preparations of Calibration Standards, Internal Standards, and Antioxidants

Stock solutions of oxylipin chemical standards were prepared at a concentration of 100 ng/μL in ethanol for all analytes except 9(10)-epoxyoctadecenoic acid, prostaglandin (PG) F1α, and 11-deoxy PGE1, which were dissolved in methanol. An intermediate stock solution containing the 43 analytes was prepared at 10 μg/μL for all standard compounds. From this intermediate stock solution, calibrator working solutions were derived through dilution with ethanol, achieving concentrations of 0.01, 0.05, 0.2, 1, 5, and 20 ng/mL for all analytes. A mixture comprising five working internal standard (IS) solutions was prepared for each individual stock of internal standard to give a final concentration of 100 ng/mL in methanol and acetonitrile (1:1, *v*/*v*), except for PGE2-d4, which was prepared at 500 ng/μL. A mixture comprising five working IS solutions was similarly prepared, with each individual stock of IS contributing to a final concentration of 100 ng/mL in a methanol and acetonitrile mixture (1:1, *v*/*v*), except for PEG2-d4, which was prepared at 500 ng/μL. To simulate the extraction of the sample compounds, five deuterated compounds (PGE2-d4, PGF2α-d4, 13-hydroxyoctadecadienoic acid-d4, 20-HETE-d8, and 5-HETE-d8) served as ISs and were added to biopsies prior to sample preparation. The selection of an appropriate IS for each analyte was based on structural similarities and retention time. To prepare the antioxidant solution, equal weights of butylated hydroxytoluene and ethylenediaminetetraacetic acid were dissolved in a methanol and distilled water mixture (1:1, *v*/*v*) to achieve a final concentration of 0.2 mg/mL.

### 2.12. Solid Phase Extraction (SPE) of Oxylipins from Biopsy Sample

Equal quantities (8 ± 1 mg) of vulvar biopsy samples were weighed and placed in a 2 mL screw cap tube containing three magnetic beads and 270 µL of extraction solution (250 μL methanol, 10 μL antioxidant solution, and 10 μL IS), followed by 1 min of homogenization in a Tissue Lyser II (QIAGEN, Hilden, Germany). Then, the supernatant was isolated by centrifugation at 12,000 rpm (4 °C) for 20 min and transferred to a 5 mL screw cap prior to solid phase extraction (SPE). Oxylipins were extracted using Oasis HLB cartridge columns (30 mg, 1 cc, Waters, Wilmslow, UK) [28]. The extraction protocol involved an initial washing step with 6 mL methanol (MeOH), followed by equilibration using 6 mL of 5% MeOH in 0.1% acetic acid. Upon sample loading, impurities were removed through a wash with the 5% MeOH and 0.1% acetic acid mixture. The target metabolites were eluted using 4 mL of methanol and subsequently transferred to 5 mL Eppendorf tubes. The eluent was dried under vacuum and stored at −80 °C. The dried pellet was reconstituted in 100 μL of a MeOH/acetonitrile mixture (50:50, *v*/*v*). The resolubilized material was transferred to a 1.5 mL microfuge tube and subjected to centrifugation at 12,000 rpm (4 °C) for 20 min. A portion (60 μL) of the supernatant was transferred into a sampling vial for subsequent oxylipin mass spectral analysis [29].

### 2.13. Liquid Chromatography–Tandem Mass Spectrometry (LC–MS/MS) Analysis

The LC–MS/MS analysis employed an ultra-performance liquid chromatographic system (UPLC, Agilent 1260, Santa Clara, CA, USA) and electrospray ionization (ESI) on a triple quadrupole mass spectrometer (QqQ, Agilent 6460C, Santa Clara, CA, USA). A 10 μL portion of the extract was injected for analysis, with the autosampler maintained at 6 °C. Separation of analytes was accomplished using an Agilent Poroshell EC-C18 column (3.0 × 150 mm; 1.9 µm; Agilent, Santa Clara, CA, USA). Mobile phase A consisted of water and acetic acid (100:0.1 *v*/*v*), while mobile phase B was composed of acetonitrile/isopropanol (90:10, *v*/*v*). The flow rate at 40 °C was maintained at 0.5 mL/min. The elution gradient conditions were as follows: 10–35% B from 0 to 3.5 min, 35–40% B from 3.5 to 5.5 min, 40–42% B from 5.5 to 7 min, 42–50% B from 7 to 9 min, 50–65% B from 9 to 15 min, 65–75% B from 15 to 17 min, 75–85% B from 17 to 18.5 min, 85–95% B from 18.5 to 19.5 min, 95–10% B from 19.5 to 21 min, and held at 10% B from 21 to 25 min. The electrospray ionization (ESI) was operated in negative ion mode, with the following parameters: sheath temperature at 350 °C, sheath gas flow at 10 L/min, dry gas temperature at 230 °C, drying gas flow at 11 L/min, nebulizer pressure at 35 psig, capillary voltage at 3500 V, nozzle voltage at 1250 V, and operating in dynamic Multiple Reaction Monitoring (dMRM) scan mode. Oxylipins were identified using the parent and product ions within the expected retention time (within 1–2 min). The parent ion was used for quantification, and the other two product ions were used to confirm the identification. The compound concentration quantification was achieved with a calibration standard curve using QQQ Quantitative Analysis software (Quant-My-Way™, version 11.0, Agilent, Santa Clara, CA, USA) [24].

### 2.14. Machine Learning Development and Validation

To select the most effective binary classification models for tape stripping and biopsy sampling, seven machine learning methods were evaluated, including artificial neural network (ANN), decision tree (DT), K nearest neighbor (KNN), naïve bayes (NB), logistics regression (LR), random forest (RF), and support vector machine (SVM). The workflow of machine learning is illustrated in Figure 1d. After the metabolomic data were scaled by log_2_ transformation and z-score normalization, the dataset was randomly regrouped into the training and testing datasets. The significant features were identified by recursive feature elimination (RFE) methods from the training dataset. These selected features were then used in training seven supervised machine learning models to establish reliable classifiers using R packages (R version 4.1.1; The R Foundation for Statistical Computing, Vienna, Austria), such as Caret, neuralnet, e1071, kknn, and C50 [30,31,32,33,34]. Subsequently, the model was internally validated using a stratified 5-fold cross-validation approach through hyperparameter tuning for each model with the testing datasets. Feature importance gain was utilized to determine the significance ranking of features in each model [35]. In addition, an independent cohort was collected to further validate the performance of the best machine learning model. The significant ranking features identified from initial models were employed to perform the external validation of the independent dataset for machine learning algorithms. Metrics including true positive (TP), true negative (TN), false positive (FP), and false negative (FN) were computed for performance evaluation as follows [36]:

       Accuracy = (TP + TN) = (TP + FP + TN + FN);

Sensitivity = TP = (TP + FN);

Specificity = TN = (TN + FP);

F1             

The discrimination ability of the models was displayed through receiver operating characteristic (ROC) curves. The area under the curve (AUC) was used to determine the sensitivity and specificity of the predicted models, where a higher AUC value signifies the superiority of the classifier [37].

### 2.15. ELISA

A direct enzyme-linked immunosorbent assay (ELISA) technique was employed, using kits from Ruixin Biotech (Quanzhou, China) for Human PLA2, Human arachidonate 5-lipoxygenase (ALOX5), Human transient receptor potential cation channel subfamily V member 1 (TRPV1), and Human pyruvate kinase isoform M2 (M2-PK) and from UpingBio (Wuhan, China) for Human cytochrome P450 family 4 subfamily a member 11 (CYP4A11) and Human tumor necrosis factor-α (TNF-α), following the manufacturers’ protocols. Protein standard solutions (50 μL) were dispensed into the standard wells, while each skin tissue sample (50 μL) was added to the assay wells on a 96-well microwell plate. Subsequently, 100 μL of specific labeled primary antibodies were added. After incubating for 60 min at 37 °C in a light-protected environment, the wells were washed, and a substrate mixture of 0.01% H_2_O_2_ with 0.1% TMB (1:1, *v*/*v*) was added (100 μL per well). This was followed by a further incubation under light-protected conditions at 37 °C for 15 min. The reaction was terminated, and the optical density (OD) at 450 nm was quantified using a spectrophotometer. The OD_450_ values were analyzed using a standard curve to establish the protein concentrations.

### 2.16. Statistical Analysis

Partial least squares discriminant analysis (PLSDA) was performed to compare global metabolomic profiles among four groups (LSC tape, NOR tape, LSC biopsy, and NOR biopsy) employing the MassOmics R package (Chongqing, China) [26]. Student’s *t*-test was used to determine whether the concentration of each identified metabolite was significantly different between LSC and NOR groups for both tape and biopsy samples using R. Subsequently, false discovery rates (FDR) were calculated for metabolites to correct for multiple tests using the q-value R package (Vienna, Austria) [38]. Results with a *p*-value < 0.05 and corresponding FDR < 0.05 were considered statistically significant. Volcano plots were generated using the OmicStudio tools (https://www.omicstudio.cn/tool, accessed on 22 July 2024). Boxplots were constructed using three R packages: ggplot2, ggpubr, and ggbeeswarm (Vienna, Austria), while the heatmap was constructed using ggplot2 [39,40,41]. Metabolic pathways were estimated using KEGG metabolic pathways, and their metabolic activities were illustrated by a bubble plot using ggpolt2, Hmisc, and scales R packages (Vienna, Austria) [42]. The Sankey diagram connecting metabolites and their participating metabolic pathways was constructed using the OmicShare tool (https://www.omicshare.com/tools, accessed on 22 July 2024). Linear regression was performed to analyze the relationship between significant biopsy metabolite concentrations and Cattaneo scores. Finally, a scatter plot, including trend lines and data markers, was created using the ggplot2 R package (Vienna, Austria) [43].

## 3. Results

### 3.1. Clinical Characteristics

The demographic and clinical characteristics of the initial 36 participants are described in Table S2. The average age of the participants was approximately 42 years with a standard deviation of 12. The Cattaneo scores had a mean value of 9.2 (±1.8). Gestation and pregnancy averages were 3 (±2) and 1 (±1), respectively. The mean duration of LSC was 39.5 (±49.9) months (ranging from 2 to 240), and the BMI showed an average value of 23.9 (±4.1).

### 3.2. Morphological Evaluation of Skin Biopsy Samples for LSC

The histopathological examination of the vulvar biopsy samples (LSC Biopsy) revealed characteristic features consistent with LSC, as shown in Figure 1e. Compared to the NOR biopsy, the LSC biopsy displayed severe acanthosis, elongation of the rete ridges, and extensive hyperkeratosis with compact orthokeratosis, indicating substantial thickening of the stratum corneum. Additionally, the granular and stratum spinosum layers were thickened, accompanied by chronic lymphocytic infiltration into the superficial dermis. These observations underscore the proliferative and inflammatory nature of LSC, corroborating the clinical diagnosis.

### 3.3. Overview of Changes in Metabolite Levels

**Figure 2:**
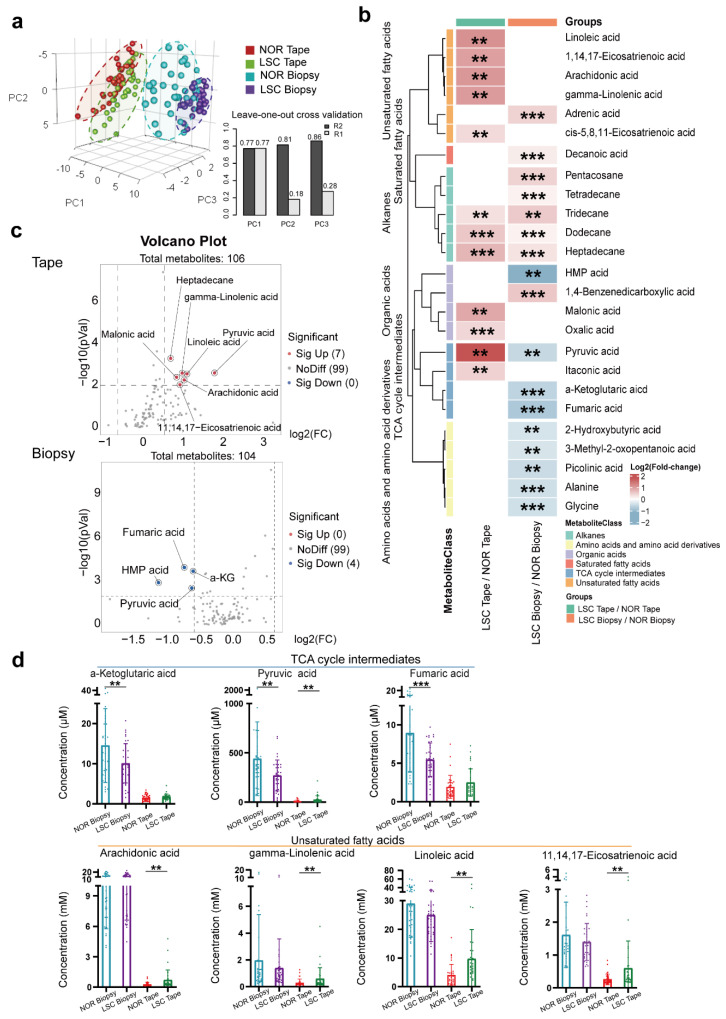
Metabolite profiles of tape stripping and biopsy samples in the LSC and normal skin groups (n = 36). (**a**) Partial Least Squares Discriminant Analysis (PLSDA): LSC tape stripping (green), normal tape stripping (red), LSC biopsy (purple), and normal biopsy (blue). Leave-One-Out Cross Validation (LOOCV) analysis of the PLSDA model with good predictive performance for PC1 (R2: 0.77, Q2: 0.77), PC2 (R2: 0.81, Q2: 0.18), and PC3 (R2: 0.86, Q2: 0.28). (**b**) Heatmap showing relative metabolite concentrations for the two types of samples and their metabolic classification. The relative concentrations of sample metabolites are illustrated using log2 scale. Red color blocks represent higher metabolite levels in numerator (LSC samples) than in the denominator (normal samples), whereas blue color blocks represent lower metabolite levels in LSC samples than in the normal samples. Only the metabolites with a *p*-value (Student *t*-test) less than 0.01 and a q-value (false discovery rate, FDR) less than 0.05 are displayed. (**c**) Volcano plot of the metabolites with differential concentrations (*p* < 0.05, FC > 1.5) between LSC and normal samples in the biopsy and tape stripping groups. Red dots indicate increased concentrations, while blue dots indicate decreased concentrations in LSC samples compared to the normal samples. (**d**) Box plots of actual metabolite concentrations for TCA cycle intermediates and unsaturated fatty acids. (** *p*-values < 0.01, *** *p*-values < 0.001). Abbreviations: LSC, lichen simplex chronicus; NOR, normal; HMP acid, 3-(4-hydroxy-3-methoxyphenyl)-,2-propenoic acid; and a-KG, a-Ketoglutaric acid.

A total of 104 and 106 metabolites were identified in vulvar skin biopsy and tape stripping samples, respectively, using the in-house mass spectrometry library and the NIST commercial database. We performed a PLSDA depicted in Figure 2a, which demonstrated a clear separation between the LSC and NOR groups of biopsy and tape samples. However, tape stripping samples showed a less pronounced difference between the LSC and NOR groups.

### 3.4. Different Metabolite Levels in Biopsy and Tape Samples

To examine the metabolic variations between the biopsy and tape groups, we utilized heatmaps, volcano plots, and box plots, as illustrated in Figure 2b–d. This analysis identified an increase in the concentration of eight metabolites and a decrease in nine metabolites in the LSC biopsy samples compared to normal skin. Notably, these differentially abundant metabolites were predominantly TCA cycle intermediates, amino acids and their derivatives, fatty acids (both saturated and unsaturated), organic acids, and alkanes. In contrast, the LSC tape samples exhibited elevations in 11 metabolites relative to normal skin samples, which mainly consisted of fatty acids and alkanes. To further elucidate the patterns of these differentially expressed metabolites in both sampling types, we performed volcano plot analyses, as presented in Figure 2c. In the LSC tape group, pyruvic acid, 11-eicosenoic acid, and linoleic acid were found to be significantly upregulated (*p* < 0.05) with fold changes (FC) exceeding 1.5. Conversely, the LSC biopsy group exhibited significant downregulation in metabolites including (3-(4-hydroxy-3-methoxyphenyl)-,2-propenoic acid), α-ketoglutaric acid, fumaric acid, pyruvic acid, and benzenepropanoic acid. The concentration distributions of these differentially abundant metabolites are displayed in Figure 2d.

### 3.5. Machine Learning Algorithms for Disease Prediction

**Figure 3:**
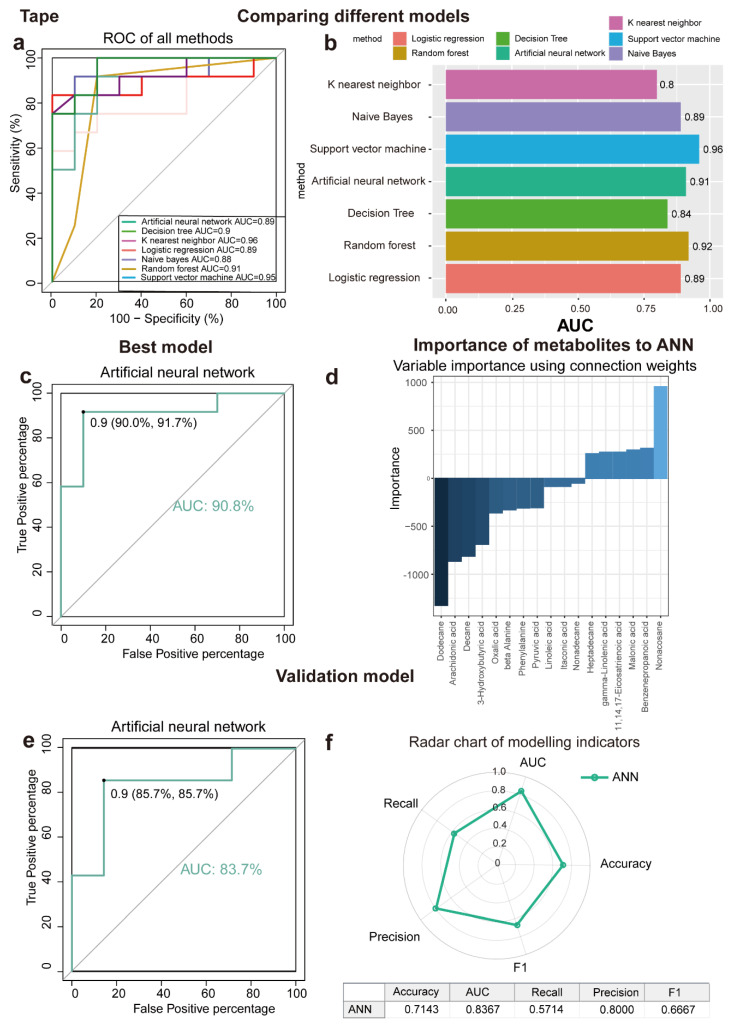
Seven machine learning models to identify discriminating metabolites for tape samples. (**a**,**b**) ROC curve analysis and corresponding AUC (median with 95% confidence interval) of every model for tape samples. (**c**) The AUC of ANN is 90.8%. (**d**) Importance of metabolites to ANN model. (**e**) ANN is the best model validation (AUC = 83.7%) using an independent patient cohort. (**f**) Radar chart illustrating the performance of the ANN model across key metrics, including accuracy, AUC, recall, precision, and F1 score. A larger area enclosed by the chart signifies better overall performance across multiple evaluation criteria. Abbreviations: ROC, receiver operating characteristic; AUC, the area under the ROC curve; ANN, artificial neural network; DT, decision tree; KNN, K nearest neighbor; LR, logistics regression; NB, Naïve Bayes; RF, random forest; and SVM, support vector machine.

**Figure 4:**
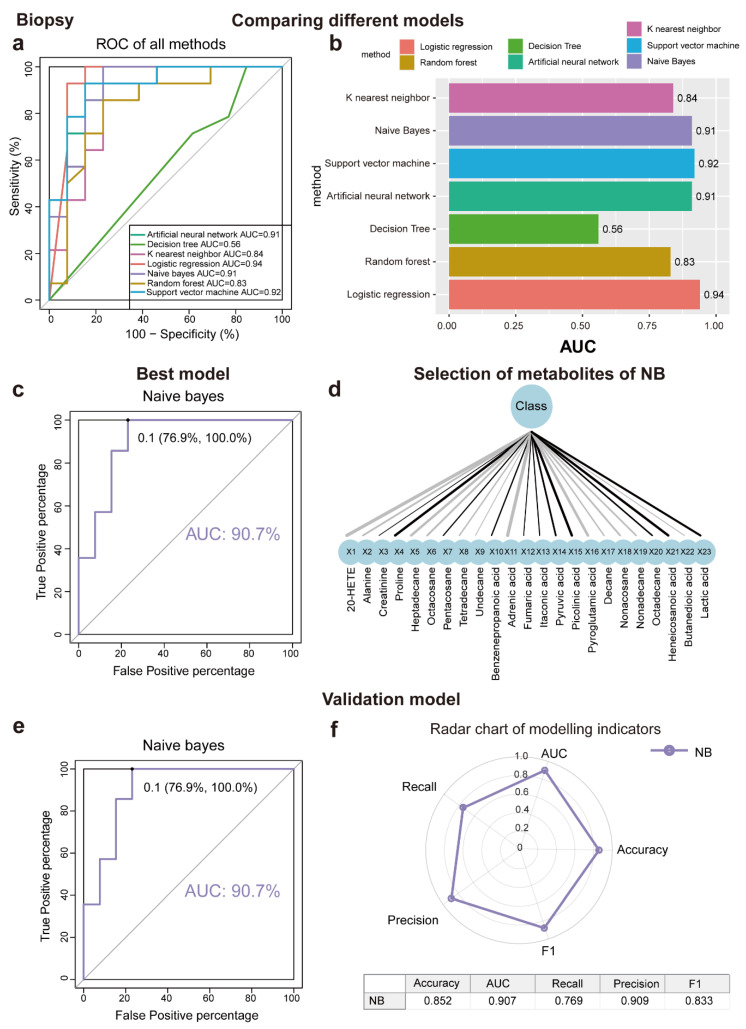
Seven machine learning models to identify discriminating metabolites for biopsy samples. (**a**,**b**) ROC curve analysis and corresponding AUC (median with 95% confidence interval) of every model for biopsy samples. (**c**) NB is the best machine learning model with AUC = 88.9%. (**d**) Selection of metabolites for NB model using recursive feature elimination (RFE). (**e**) NB model validation (AUC = 90.7%) using an independent patient cohort. (**f**) Radar chart illustrating the performance of the NBD model across key metrics, including accuracy, AUC, recall, precision, and F1 score. A larger area enclosed by the chart signifies better overall performance across multiple evaluation criteria. Abbreviations: ROC, receiver operating characteristic; AUC, the area under the ROC curve; ANN, artificial neural network; DT, decision tree; KNN, K nearest neighbor; LR, logistics regression; NB, Naïve Bayes; RF, random forest; and SVM, support vector machine.

This study aimed to determine metabolites that can discriminate between LSC skin and normal skin, various algorithms, including ANN, DT, KNN, LR, NB, RF, and SVM, were implemented. As a result of applying the machine learning algorithms to the classification of samples, a panel of 17 metabolites in tape samples and 23 in biopsy samples was identified. The contributions of these metabolites to the performance of each machine learning algorithm were ranked (Figure 3a,b and Figure 4a,b). The biomarker signatures discriminating the LSC group from the normal groups in tape and biopsy are displayed in Figure 3d and Figure 4d. In biopsy samples, NB (AUC = 0.91), SVM (AUC = 0.92), ANN (AUC = 0.91), and LR (AUC = 0.94) exhibited the highest AUC values (Figure 4a). Conversely, in tape samples, ANN (AUC = 0.91), SVM (AUC = 0.96), and RF (AUC = 0.92) demonstrated the highest AUC values (Figure 3a). To further validate the performance of the top-ranked features, model validation was conducted using independent samples from an additional 11 patients. NB (AUC = 0.91) exhibited the best independent validation compared to other models (SVM, AUC = 0.67; ANN, AUC = 0.83; and LR, AUC = 0.56) in biopsy samples, while ANN (AUC = 0.84) demonstrated the best independent validation compared to other models (SVM, AUC = 0.74; and RF, AUC = 0.68) in tape samples (Figure 3e, Figure 4e and Figure S1). Our machine learning analysis suggests that ANN is well-suited for tape samples, demonstrating superior performance. Conversely, NB emerged as the best model for biopsy data, especially given its exceptional results in the external validation process. Noticeably, the metabolite levels detected in tape samples were generally lower compared to those in biopsies. (Figure 2d); despite this, tape samples could be used to identify LSC cases with reasonable accuracy.

### 3.6. Metabolic Pathway Enrichment Analysis

**Figure 5:**
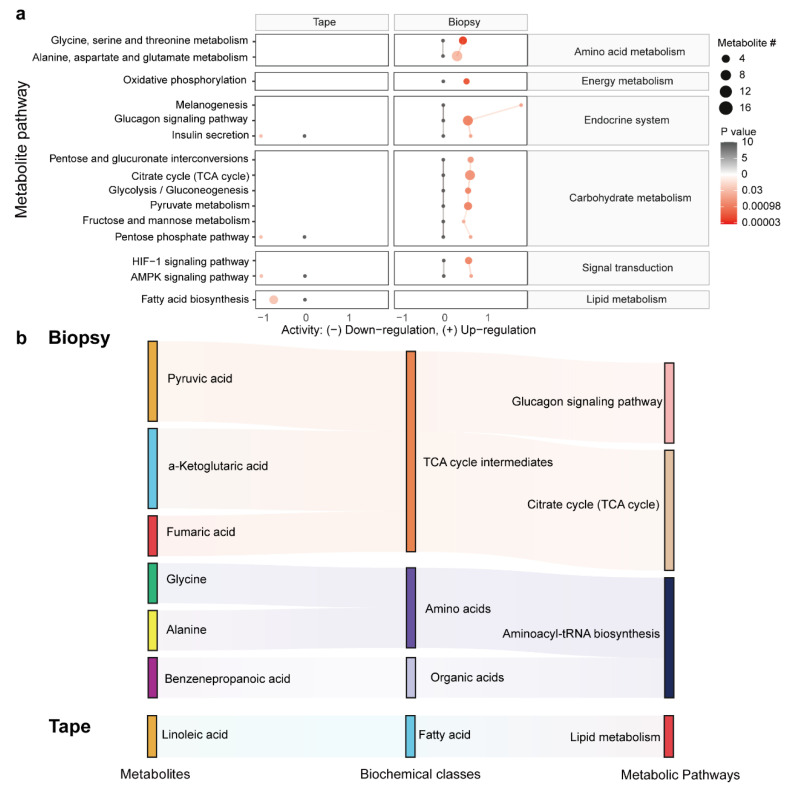
The association between metabolic pathways and differentially abundant metabolites. (**a**) The predicated activities of metabolic pathways derived from the metabolome of biopsy and tape stripping samples. Black dots represent the adjusted metabolic activities in the normal group (set to 0), while red dots represent levels in the LSC group compared to the normal group. The differences in metabolite levels are represented using a log2 scale. The dot size corresponds to the pathway’s metabolite count, whereas its color reflects the *p*-value. (**b**) A Sankey diagram visually representing the connections between metabolites (left), their biochemical classes (middle), and various metabolic pathways (right) in both biopsy and tape stripping samples, offering a comprehensive view of how metabolites are linked to different metabolic pathways.

To further explore the biological role of differentially abundant metabolites, we performed a pathway enrichment analysis based on the KEGG metabolic network (Figure 5a). The predicted pathway analysis showed that a predominant portion of the differences in the metabolism of the tissues encompass energy metabolism, the endocrine system, carbohydrate metabolism, signal transduction, and amino acid metabolism, which were upregulated in the LSC biopsy. In contrast, four metabolic pathways associated with the metabolism of the endocrine system, signal transduction, and lipid biosynthesis were downregulated in the LSC tape samples. Next, we took the metabolites identified by the machine-learning models and linked them to the altered metabolic pathways using a Sankey diagram (Figure 5b). The majority of metabolites from the biopsy samples participated in the TCA cycle, whilst a reduction in linoleic acid was linked to the decreased lipid metabolism observed in the tape samples.

### 3.7. Correlation Between LSC-Related Metabolites and Severity of LSC (Cattaneo Scores)

**Figure 6:**
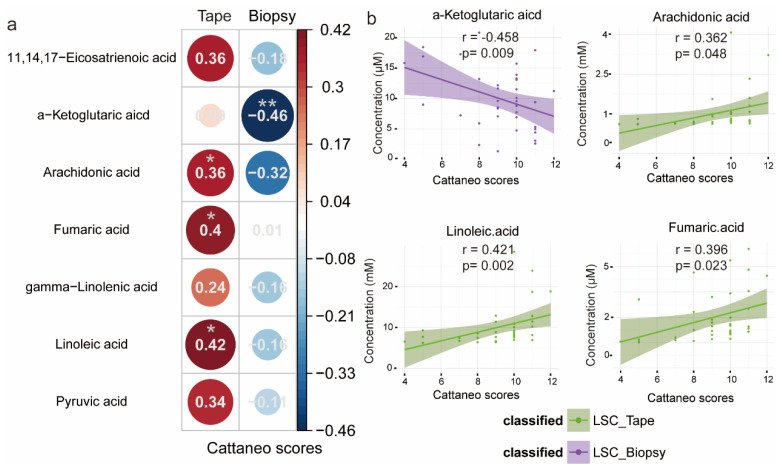
Standardized linear regression analyses displaying the relationship between differentially abundant metabolites and the LSC Cattaneo scores for the tape and biopsy groups. (**a**) Circular correlogram showing relationships between Cattaneo scores and metabolite levels. Numbers and circle sizes represent standardized regression coefficients. Red and blue circles indicate positive and negative linear associations, respectively (* *p*-values < 0.05, ** *p*-values < 0.01). (**b**) Scatter plots with regression lines for metabolites significantly correlated with Cattaneo scores. The shadow around the trendline shows the 95% CI. r is the standardized regression coefficient, and *p* is the statistical significance.

For both tape and biopsy samples, we conducted a standardized linear regression analysis to investigate the relationship between differentially abundant LSC metabolites and the Cattaneo scores, as depicted in Figure 6. The standardized coefficients, indicating the effect of one standard Cattaneo score change on the standardized metabolite concentration, revealed a negative correlation between the significant LSC metabolites and the Cattaneo scores for the LSC biopsy samples. Conversely, a positive association between the Cattaneo scores and the metabolite concentrations was observed for the LSC tape samples. In biopsy samples, alpha-ketoglutarate exhibited a significant negative correlation, while in tape samples, AA, LA, and fumaric acid showed a significant positive correlation. The results suggest that this metabolite in biopsy samples is inversely associated with the severity of LSC, offering further insights into potential markers for the diagnosis of LSC.

### 3.8. A Derive Oxylipin Profiling of LSC

**Figure 7:**
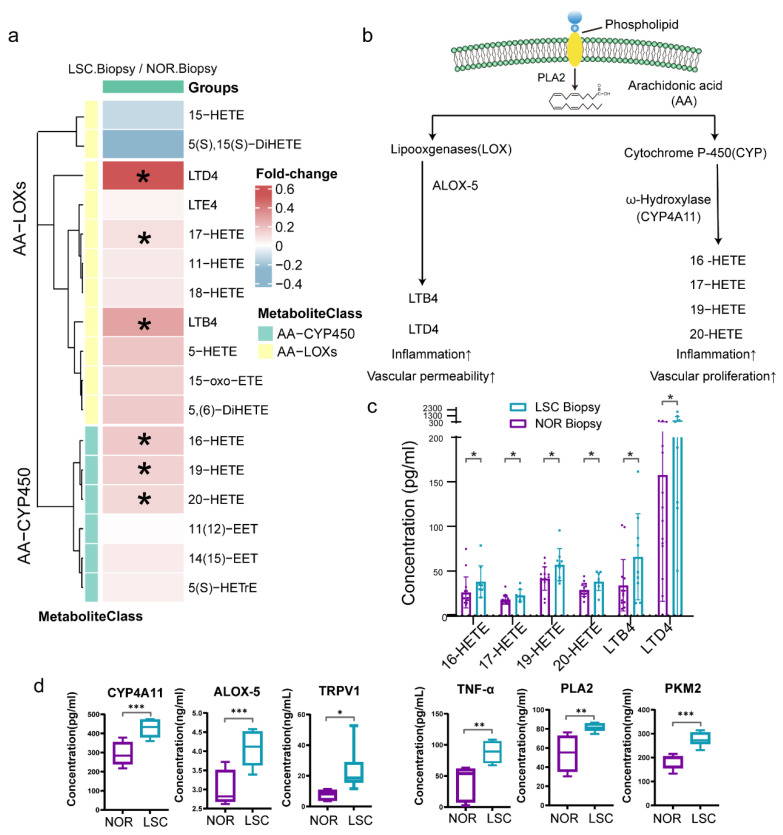
Oxylipin profiling of biopsy samples. (**a**) Heatmap showing concentrations of oxidized lipid metabolites in biopsy samples relative to normal samples and their associated metabolic classification. (**b**) Schematic diagram of the lipid oxidation metabolic pathway. (**c**) Boxplots of actual oxidized lipid metabolite concentrations in panel b. (**d**) Boxplots of protein levels measured by ELISA. * *p*-values < 0.05, ** *p*-values < 0.01, *** *p*-values < 0.001. Abbreviations: LTB4, Leukotriene B4; LTD4, Leukotriene D4; HETE, Hydroxyeicosatetraenoic acid; PLA2, Phospholipase A2; CYP4A11, Cytochrome P450 4A11; ALOX-5, Arachidonate 5-lipoxygenase; TNF-α, Tumor Necrosis Factor-alpha; TRPV1, Transient Receptor Potential Vanilloid 1; and PKM2, Pyruvate Kinase M2.

To investigate the downstream products of omega-6 fatty acids derived from linoleic acid and arachidonic acid, we also quantified 20 oxylipins in samples from both LSC and NOR groups (Figure 7a). There were elevated concentrations of the majority of oxylipins in LSC samples, particularly the downstream oxylipins of arachidonic acid-lipoxygenases (AA-LOX) and arachidonic acid-cytochrome P450 (AA-CYP450). Importantly, downstream oxylipins of AA-LOX, namely LTB4 and LTD4, are associated with inflammation and vascular permeability. The AA-CYP450-catalyzed metabolism produced n-HETE analogues (16-HETE, 17-HETE, 18-HETE, 19-HETE, and 20-HETE), which contribute to inflammatory responses and vascular endothelial cell proliferation (Figure 7b,c) [44,45]. However, no statistically significant differences in concentrations were observed for the downstream oxylipin products of linoleic acid. To substantiate the changes in metabolite levels, the expression of pivotal enzymes and proteins involved in lipid oxidation and energy metabolism in tissue samples from five individuals was measured by ELISA. We found an upregulation in the expression of enzymes and inflammatory factors, including PLA2, ALOX5, CYP4A11, PKM2, TNF-α, and TRPV1, within LSC tissues (Figure 7d).

## 4. Discussion

Although alterations in skin metabolism are recognized to contribute to several chronic dermatological conditions, the specific metabolic shifts associated with LSC symptoms remain underexplored. In this study, we conducted a comparative metabolic analysis using skin secretion samples acquired via tape stripping and skin tissue biopsies from thirty-six female patients diagnosed with LSC, aiming to identify potential biomarkers for the disease and elucidate its pathogenesis. Our findings revealed significant elevations in LA and AA concentrations in skin lesions sampled by tape stripping, alongside reductions in both pyruvate and α-ketoglutarate levels in biopsy samples. In addition, the study by Hamers et al. showed that 20-HETE, the downstream product of AA, may interact with TRPV1 in LSC lesions, potentially contributing to pruritic symptoms [46]. Furthermore, our study indicates that elevated PKM2 expression and enhanced energy metabolism may facilitate epidermal proliferation, leading to skin thickening. Thus, our research presents a novel, non-invasive diagnostic approach utilizing tape stripping to differentiate the manifestations of LSC, capitalizing on the unique metabolic profile of LSC to investigate its intricate pathogenesis.

### 4.1. The Potential Application of Tape Stripping in LSC Diagnosis

Non-invasive or minimally invasive sampling methods have made significant advances in the diagnosis of skin diseases. Tape stripping, as a non-invasive skin sampling technique, not only minimizes patient discomfort but also mitigates the risks of infection and complications associated with traditional biopsies. We found that LA and AA obtained through tape stripping from LSC skin demonstrated outstanding discriminatory performance in an external validation using seven machine learning algorithms (Figure 3). Additionally, tape stripping has found extensive application in the field of dermatology for other conditions characterized by recurrent itching and skin lesions, including atopic dermatitis (AD) and psoriasis. Seibold et al. used tape stripping to extract RNA from the lesional skin of AD patients and found increased expression of the auxiliary T-helper 2 gene using RNA-seq [47]. Jankovskaja et al. successfully identified the phenylalanine/tryptophan ratio as a biomarker for skin cancer detection using tape stripping to collect skin samples for metabolomic analysis [48]. Another study demonstrated the capability to differentiate between psoriasis and AD of varying severity by employing a tape stripping-based approach [49]. Skin tape stripping requires no special preparation of the skin and no anesthetic, allowing for direct sampling of the skin lesion. Thus, tape stripping demonstrates promising discriminatory performance, offering a convenient and effective approach to the diagnosis of various skin conditions. However, a potential drawback of tape stripping is the difficulty in sampling standardization. Tape stripping results are influenced dramatically by various parameters, including the anatomical site, the pressure applied, the duration of pressure application, and the tape removal rate [50]. Therefore, we employed a standardized sampling protocol aimed to minimize the variability in the collection of skin specimens. A uniform pressure and adhesive time were consistently applied across all participants, with two sampling sites strictly confined to the perineal region for enhanced precision. While tape stripping offers a non-invasive alternative, it is complementary to biopsy, providing valuable insights alongside traditional methods. Overall, our results suggest that the standardized tape stripping method is able to collect skin metabolites with high reproducibility from vulvar skin. It could be considered a noninvasive and effective method for LSC skin metabolomic studies.

### 4.2. Release of LA and AA After Skin Damage in LSC

Skin lipids are pivotal for maintaining the integrity of the skin’s barrier function and modulating skin inflammation. LA and AA are omega-6 polyunsaturated fatty acids, which are free fatty acids found in the stratum corneum of human skin and are important components of skin cell membrane phospholipids [51]. Our study revealed elevated LA, AA, and PLA2 levels in the skin from LSC lesions. Research has indicated a correlation between cell membrane damage and increased in vivo PLA2 activity [52]. In addition, Sackheim et al. demonstrated a significant upregulation in PLA2 activity associated with cell membrane damage in mice [53]. Shao et al. observed an increased PLA2 expression in psoriasis and bacterial skin infections and demonstrated that silencing the PLA2 gene in mice with psoriasis significantly mitigated disease symptoms [54]. PLA2 is capable of hydrolyzing the sn-2 position of glycerophospholipids in cell membranes, releasing free fatty acids, including LA and AA [55]. Andersen et al. identified elevated levels of AA, along with overexpression of nonpancreatic phospholipase A2 (npPLA2), in the affected skin of patients with psoriasis [56]. Based on these findings, we suggest that skin damage in LSC patients leads to PLA2 activation, hydrolysis of skin cell membrane phospholipids, and the release of AA and LA.

### 4.3. The Inflammatory Activity of AA-Derived HETE and Leukotrienes in LSC

Cytochrome P450 ω-hydroxylases can catabolize AA into HETEs, including 20-HETE [45]. We found elevated concentrations of 20-HETE, LTB4, and LTD4, alongside the expression of oxylipin biosynthesis enzymes, including CYP4A11, ALOX5, and TNF-α, in the skin lesions of patients with LSC. CYP4A11 is the P450 ω-hydroxylase with the highest expression in skin tissues. Specifically, exposure to foreign substances and stimuli such as ultraviolet radiation leads to increased CYP4A11 mRNA expression in skin keratinocytes [57]. 20-HETE has been demonstrated to enhance the expression of endothelial cell activation-induced adhesion molecules and inflammatory cytokines, thereby promoting vascular inflammation [58]. 20-HETE also stimulates TNF-α secretion by activating mitogen-activated protein kinases [58,59,60]. Regner et al. reported that inhibition of 20-HETE synthesis reduced oxidative stress and vascular expression of TNFα, IL-1β, and IL-6 [61]. The proinflammatory cytokine TNF-α could also act in a feedback loop, activating cytochrome P450 ω-hydroxylase activity and PLA2, forming a vicious pathological cycle [62]. Conversely, AA is also catabolized into leukotrienes, such as LTB4, through the enzymatic activity of ALOX-5, a member of the LOX enzyme family. Studies have identified a significant upregulation of ALOX-5 expression and elevated concentrations of LTB4 in the dermal layers of patients with systemic sclerosis [63,64]. LTB4 acts as a potent chemokine that recruits neutrophils in dermatitis resulting from skin damage [65]. We propose that the activation of CYP450 ω-hydroxylase and ALOX-5, which catalyze the catabolism of AA to produce significant amounts of 20-HETE and leukotrienes in LSC lesions, contributes to the generation and exacerbation of inflammatory responses.

### 4.4. The Interplay of 20-HETE, TRPV1, and PKM2 in LSC Pathogenesis

Transient receptor potential cation channel subfamily V member 1 (TRPV1) is present in a wide range of skin cells and plays a role in itch transmission, skin barrier homeostasis, epidermal proliferation, and inflammation [66]. We found higher expression of TRPV1 in LSC lesions than in normal skin. While TRPV1 is primarily known for its role in pain perception, growing evidence suggests that it may also contribute to pruritus in some inflammatory skin conditions. Increased expression and phosphorylation of TRPV1 has been observed in atopic dermatitis and psoriasis, where it may correlate with itch severity [67,68]. Additionally, oxidized lipids such as 20-HETE have been reported to activate TRPV1 in preclinical models, inducing neuropeptide release and neurogenic inflammation [69]. Although our data show increased levels of both 20-HETE and TRPV1, we acknowledge that a direct functional link between these molecules and the itch–scratch cycle in LSC was not demonstrated in this study. Therefore, we propose a possible involvement of the 20-HETE–TRPV1 axis in LSC-related pruritus, which warrants further functional validation.

**Figure 8:**
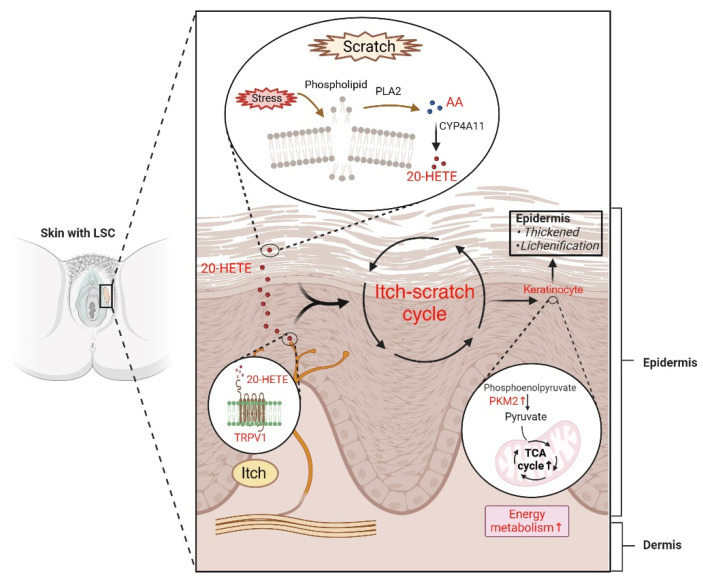
Summary of proposed metabolic mechanisms underlying lichen simplex chronicus (LSC). Vulvar itching is intricately linked to the specific anatomical location of the vulva, rendering it more susceptible to irritants due to its proximity to the urinary, reproductive, and digestive tracts. Additionally, external stress such as chafing and scratching can disrupt skin cell membranes, triggering the activation of PLA2. This enzyme catalyzes the breakdown of phospholipids in the skin into AA, which further undergoes metabolism by CYP4A11 to form 20-HETE. Furthermore, 20-HETE can directly bind to the TRPV1 receptor, inducing itching and initiating the itch–scratch cycle. Subsequent scratching enhances PKM2 activity and increases tricarboxylic acid cycle flux. Ultimately, these cascading events fuel keratinocyte proliferation and culminate in the thickening and lichenification of the vulvar skin. Abbreviations: TCA, tricarboxylic acid. (Created with BioRender.com, accessed on 7 August 2024).

The itch–scratch cycle exacerbates LSC by contributing to skin thickening. The pyruvate kinase M2 isoform (PKM2) is a glycolytic enzyme that converts phosphoenolpyruvate (PEP) to pyruvate, which enters mitochondria to participate in the tricarboxylic acid (TCA) cycle [70]. Our metabolite analysis of skin biopsy tissues revealed a reduction in pyruvate, fumarate, and α-ketoglutarate levels, which was accompanied by an increased TCA cycle activity and upregulated PKM2 expression in LSC lesion skin (Figure 8). Sych et al. demonstrated increased PKM2 expression in damaged skin, with immunohistochemistry staining indicating predominant expression in keratinocytes within highly proliferative epithelia [71]. Similar hyperproliferation is observed in psoriasis, where PKM2 is overexpressed in both patient epidermis and mouse models, promoting keratinocyte glycolytic metabolism [72]. Based on these findings, we propose that the skin damage induced by the itch–scratch cycle triggers PKM2 activation and upregulates energy metabolism, leading to epidermal hyperproliferation and ultimately resulting in lichenization (Figure 8).

This study provides insights into the pathogenesis of LSC and identifies a promising non-invasive tape-stripping diagnostic approach. Nevertheless, it is important to acknowledge several limitations. Primarily, the analysis is based on a comparison between diseased and healthy skin tissues of the same individuals, which may overlook the variability in skin metabolism and the inflammatory responses of individuals without LSC. Moreover, the metabolite composition of skin tape samples differs from that of biopsy samples, representing the distinct metabolic activities of skin surface secretions as opposed to the deeper tissue metabolism. The metabolites collected via tape stripping are minimal in quantity and may not fully represent the entire pathological state of the skin, being susceptible to external environmental factors, such as vaginal cleansers, medications, and inflammation. Additionally, the diagnostic efficacy of the identified biomarkers, LA and AA, requires further validation in larger and more heterogeneous cohorts to confirm their diagnostic reliability and applicability. The relatively small validation cohort is also a limitation, and larger prospective studies are needed to confirm these findings. The current study also lacks comparison with other pruritic or inflammatory dermatoses, which may affect the disease specificity of the findings. Despite these considerations, our research advances a non-invasive sampling method for LSC, facilitating the prospect of population-based validation.

## 5. Conclusions

Our study revealed that the tape stripping method offers a promising non-invasive diagnostic technique for LSC. In addition, we propose that the potential biomarkers, including AA and its downstream metabolite 20-HETE, are involved in the itch–scratch cycle by activating the TRPV1 pathway. This activation subsequently stimulates energy metabolism and contributes to lichenization. Our findings offer novel insights into the pathogenesis of LSC and suggest innovative avenues for the development of non-invasive diagnostic tools for this dermatological condition.

## Data Availability

The raw data supporting the conclusions of this article will be made available by the authors, without undue restriction.

## Supplementary Materials

The following supporting information can be downloaded at: https://www.mdpi.com/article/10.3390/metabo15090566/s1, Figure S1: The other validation models for tape strips and biopsy.; Table S1: The Cattaneo score of symptoms and signs; Table S2: Clinical characteristics of LSC patients.
