# Supplementary material for: Unraveling the Metabolic Mechanisms and Novel Biomarkers of Vulvar Lichen Simplex Chronicus Using Skin Biopsy and Tape Stripping Samples
Source: Metabolites. 2025 Aug 22;15(9):566. doi: 10.3390/metabo15090566 (PMC12472105; doi:10.3390/metabo15090566)
Supplement: Supplementary file 1 [file metabolites-15-00566-s001.zip › Figure S1 Caption.pdf]

Supplementary Figure S1: The other validation models for tape strips and biopsy.

a) Validation model for tape: The AUC of SVM and RF are 73.5% and 68.4%, respectively. b) Validation model for biopsy: The AUC of SVM is 67.3%, LR is 55.9% and ANN is 82.7%. Abbreviations: ROC, receiver operating characteristic; AUC, the area under the ROC curve; ANN, artificial neural network; LR, logistics regression; RF, random forest; and SVM, support vector machine.
