# Supplementary material for: Unraveling the Metabolic Mechanisms and Novel Biomarkers of Vulvar Lichen Simplex Chronicus Using Skin Biopsy and Tape Stripping Samples
Source: Metabolites. 2025 Aug 22;15(9):566. doi: 10.3390/metabo15090566 (PMC12472105; doi:10.3390/metabo15090566)
Supplement: Supplementary file 1 [file metabolites-15-00566-s001.zip › Table S1.pdf]

Supplementary Table S1 . The Cattaneo score of symptoms and signs

Score	Itching degree	Skin elasticity	Skin color	Lesion area
0	None	Normal	Normal	0
1	Mild	Slightly worse	Red	$<$ 30%
2	Medium	Thin skin	Pink	30%-50%
3	Heavy	Chapped skin	White	$>$ 50%
