# Supplementary material for: Unraveling the Metabolic Mechanisms and Novel Biomarkers of Vulvar Lichen Simplex Chronicus Using Skin Biopsy and Tape Stripping Samples
Source: Metabolites. 2025 Aug 22;15(9):566. doi: 10.3390/metabo15090566 (PMC12472105; doi:10.3390/metabo15090566)
Supplement: Supplementary file 1 [file metabolites-15-00566-s001.zip › Table S2.pdf]

Supplementary Table S2. Clinical characteristics of LSC patients

	LSC
Participants, n	36
Age, years	42.1 ± 11.5
BMI (kg/m2)	23.9 ± 4.1
Cattaneo	9.2 ± 1.8
Gravidity	3 ± 2
Parity	1 ± 1
Menopause	
Yes	8 (22.2%)
No	28 (77.8%)
Disease duration (months)	39.5 ± 49.9

BMI: body mass index.
