# Supplementary figures and images for: Unraveling the Metabolic Mechanisms and Novel Biomarkers of Vulvar Lichen Simplex Chronicus Using Skin Biopsy and Tape Stripping Samples
Source: Metabolites. 2025 Aug 22;15(9):566. doi: 10.3390/metabo15090566 (PMC12472105; doi:10.3390/metabo15090566)

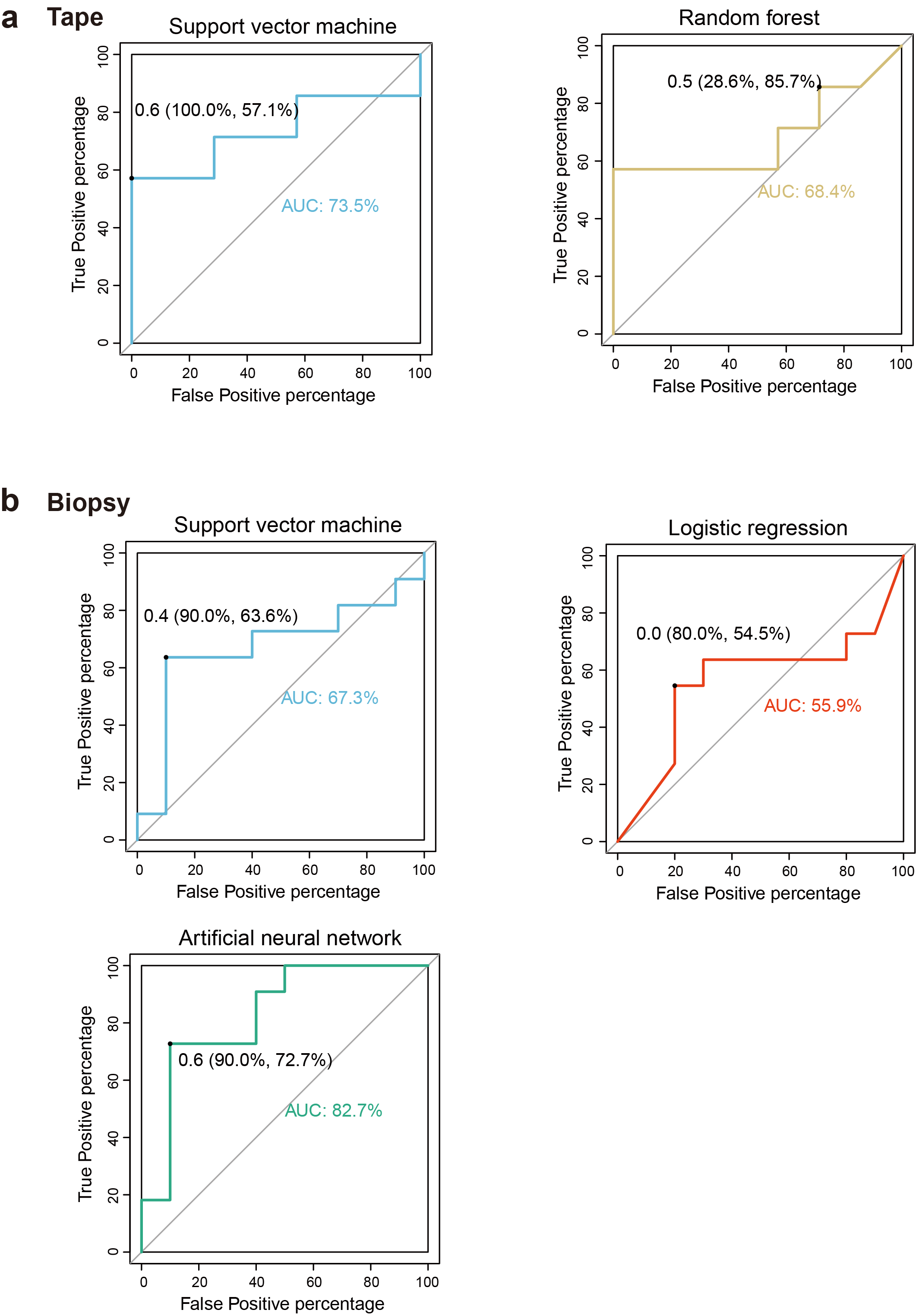

Supplement: Supplementary file 1 [file metabolites-15-00566-s001.zip › Figure S1.tif]
